# Improvements in Health‐Related Quality of Life With Treat‐to‐Target Urate‐Lowering Therapy in Gout: A Post Hoc Analysis of a Randomized Multicenter Trial

**DOI:** 10.1002/acr.25618

**Published:** 2025-11-17

**Authors:** Austin Barry, Bryant R. England, Harlan Sayles, Lindsay N. Helget, Maria Androsenko, Hongsheng Wu, Kaleb Michaud, Bridget Kramer, Jeff A. Newcomb, Mary T. Brophy, Anne Davis‐Karim, Ryan Ferguson, Michael H. Pillinger, Tuhina Neogi, Paul M. Palevsky, James R. O'Dell, Ted R. Mikuls

**Affiliations:** ^1^ VA Nebraska‐Western Iowa Health Care System and University of Nebraska Medical Center Omaha; ^2^ University of Nebraska Medical Center Omaha; ^3^ VA Boston Cooperative Studies Program Coordinating Center Boston Massachusetts; ^4^ VA Boston Cooperative Studies Program Coordinating Center and Wentworth Institute of Technology Boston Massachusetts; ^5^ VA Nebraska‐Western Iowa Health Care System and University of Nebraska Medical Center, Omaha, and Forward Databank Wichita Kansas; ^6^ VA Boston Cooperative Studies Program Coordinating Center and VA Boston Health Care System, Boston University Boston Massachusetts; ^7^ VA Cooperative Studies Program Clinical Research Pharmacy Coordinating Center Albuquerque New Mexico; ^8^ VA New York Harbor Health Care System and NYU Grossman School of Medicine New York New York; ^9^ Boston University Chobanian & Avedisian School of Medicine Boston Massachusetts; ^10^ VA Pittsburgh Health Care System and University of Pittsburgh School of Medicine Pittsburgh Pennsylvania

## Abstract

**Objective:**

Although treat‐to‐target urate‐lowering therapy (ULT) is endorsed as best practice in gout management, limited data exist on its impact on health‐related quality of life (HRQoL). We assessed the impact of treat‐to‐target ULT on HRQoL among participants receiving protocolized gout care, identifying factors associated with HRQoL and HRQoL change.

**Methods:**

This was a post hoc analysis of a 72‐week randomized trial, pooling data from allopurinol and febuxostat treatment arms. The Veterans RAND‐12 Item Health Survey and EuroQol 5‐Dimension 3‐Level (EQ‐5D‐3L) were administered at baseline and at 24, 48, and 72 weeks. HRQoL changes over follow‐up were examined using paired *t*‐tests. Factors associated with baseline HRQoL were evaluated using multivariable linear regression. General estimating equations were used to identify determinants of HRQoL change over follow‐up.

**Results:**

Participants (N = 878) in this analysis were 98.9% male, had a mean age of 62.4 years, and 67.4% self‐reported White race. HRQoL scores overall, and particularly the domains of physical function, mobility and pain, improved significantly over 72 weeks (*P* < 0.001) with improvements noted by 24 weeks. Poorer enrollment HRQoL was associated with younger age, non‐White race, tophi (for EQ‐5D‐3L), higher serum urate level, and greater comorbidity. Baseline factors associated with HRQoL improvements over 72 weeks of ULT included lower C‐reactive protein level and lower comorbidity scores with similar changes observed by ULT assignment.

**Conclusion:**

Treat‐to‐target ULT in gout is accompanied by HRQoL improvements evident by 24 weeks and sustained through 72 weeks. HRQoL gains with treat‐to‐target ULT are most prominent in the domains of physical function, mobility, and pain and are greatest in those with lower baseline levels of inflammation and comorbidity.

## INTRODUCTION

Gout is characterized by acute episodes of arthritis (or flares) separated by intercritical periods and is the most common form of inflammatory arthritis, affecting up to 5% of the population and nearly 12 million people in the United States alone.^1^ Consequently, gout is associated with significant reductions in health‐related quality of life (HRQoL) and work participation.^2^, ^3^, ^4^, ^5^, ^6^ Recognizing the detrimental impact that gout exerts on HRQoL, the Outcome Measures in Rheumatology consortium has endorsed the routine assessment of HRQoL as a patient‐reported outcome in trials investigating the efficacy of urate‐lowering therapy in gout.^7^

**SIGNIFICANCE & INNOVATIONS**

- Despite being recommended in clinical practice guidelines, the impact of treat‐to‐target urate‐lowering therapy on health‐related quality of life (HRQoL) in gout is poorly understood.
- Younger age, non‐White race, tophi, higher serum urate, and comorbidity were associated with poorer HRQoL in trial participants with gout at enrollment.
- Treat‐to‐target urate‐lowering therapy led to significant improvements in HRQoL over 72 weeks, gains that were evident as early as 24 weeks.
- The physical component score of the Veteran RAND 12‐item and EuroQol 5‐Dimension 3‐Level domains of pain and mobility demonstrated significant improvements with treat‐to‐target urate‐lowering therapy.
- Participants with gout accompanied by fewer baseline comorbidities and lower C‐reactive protein levels were most likely to demonstrate HRQoL improvements with treat‐to‐target urate‐lowering therapy.

Acknowledging the central role of hyperuricemia in gout pathogenesis, urate‐lowering therapy is a cornerstone in gout treatment and demonstrates beneficial effects that include the lowering of serum urate concentration, promoting the dissolution of monosodium urate crystals, reducing or eliminating gout flares, resolving tophi, and preventing joint damage. International subspecialty societies, including both the American College of Rheumatology (ACR) and EULAR, recommend a treat‐to‐target approach to urate‐lowering therapy with a goal of achieving and maintaining a serum urate of <6 mg/dL.^8^, ^9^ To date, most studies linking gout with reductions in HRQoL have been cross‐sectional in design.^2^ Thus, despite the well‐documented associations of gout with reduced HRQoL and published recommendations strongly endorsing treat‐to‐target urate‐lowering therapy, there remain only limited data exploring how this best‐practice management approach influences HRQoL outcomes.^10^, ^11^, ^12^

To further assess the HRQoL changes that accompany treat‐to‐target urate‐lowering therapy, we conducted a post hoc analysis of the STOP Gout trial (ClinicalTrials.gov identifier NCT02579096), a comparative effectiveness study that examined the efficacy of allopurinol and febuxostat with both agents administered following a treat‐to‐target protocol.^13^ In this analysis, data from the allopurinol and febuxostat treatment arms were pooled to test the hypothesis that study participants with gout receiving treat‐to‐target urate‐lowering therapy would yield significant improvements in HRQoL over follow‐up as assessed by the Veterans RAND 12‐item (VR‐12) and the EuroQol 5‐Dimension 3‐Level (EQ‐5D‐3L) questionnaires. We also sought to identify participant characteristics, including urate‐lowering therapy treatment assignment, that might predict such improvements, by testing the additional hypotheses that the improvement in HRQoL measures would be associated with previously documented reductions in gout flares and the achievement of serum urate goal.

## PATIENTS AND METHODS

### Data source

**Figure 1:**
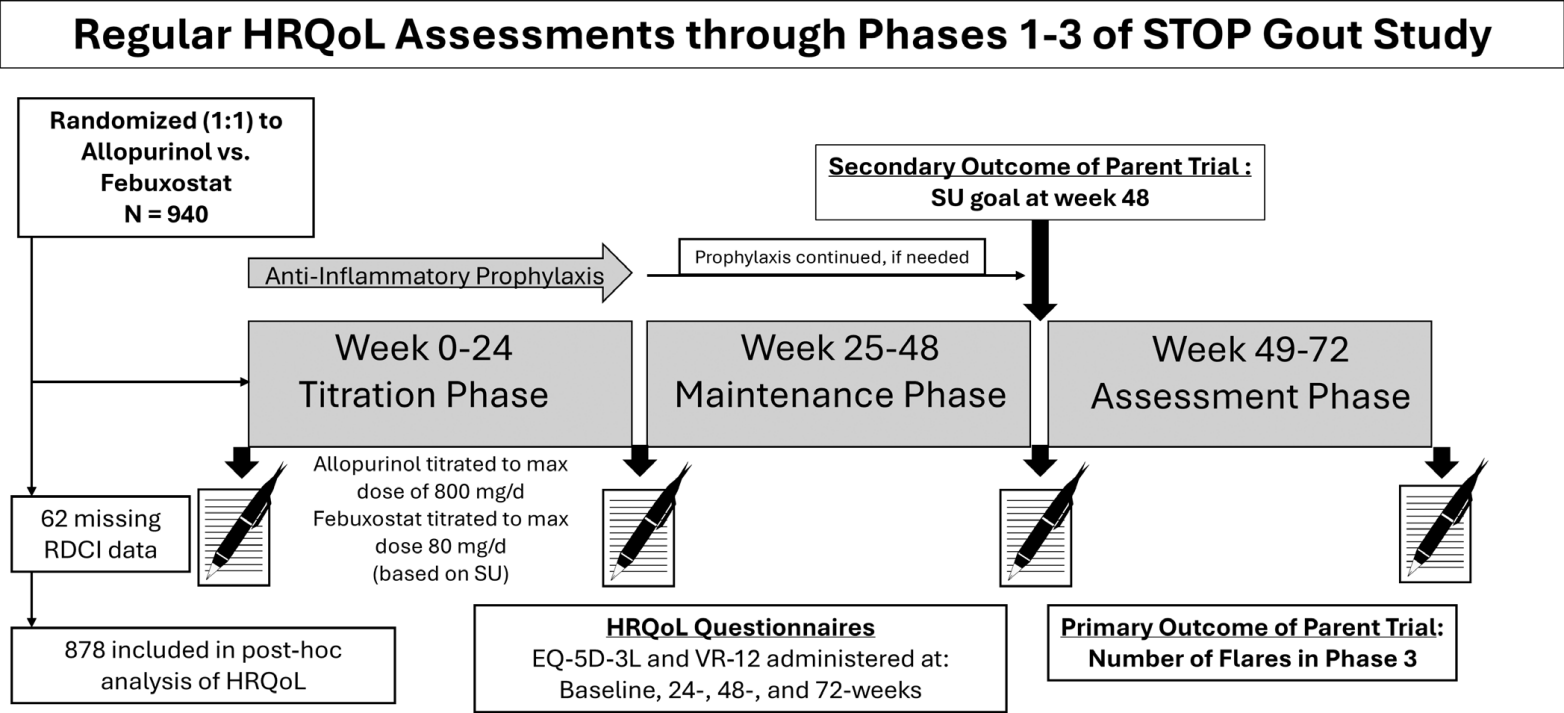
General structure and endpoints of the STOP Gout study with routine HRQoL assessments. During phase 1 (weeks 0–24), urate‐lowering therapy was titrated according to 2012 American College of Rheumatology guidelines. In phase 2 (weeks 25–48), the urate‐lowering therapy dose was adjusted as needed. In phase 3 (weeks 49–72), the urate‐lowering therapy dose was stable, and participants were monitored for flare. EQ‐5D‐3L, EuroQol 5‐Dimension 3‐Level; HRQoL, health‐related quality of life; RDCI, Rheumatic Disease Comorbidity Index; SU, serum urate; VR‐12, Veterans RAND 12‐item.

The STOP Gout study was a multicenter, randomized, double‐blind, placebo‐controlled, 72‐week noninferiority trial comparing allopurinol with febuxostat in 940 participants with a primary outcome of flare occurring during the final study phase (weeks 49–72).^13^ The trial was conducted at 19 Veterans Affairs (VA) sites and two academic medical centers. By design, approximately one‐third of participants had Stage 3 chronic kidney disease (CKD). Individuals with more advanced CKD (stages 4 or 5) were excluded. Participants with prior allopurinol use (≤300 mg/day) but not at serum urate goal were also included in the trial. The treatment protocol consisted of three phases of equal duration: urate‐lowering therapy initiation and titration (phase 1: weeks 0–24), maintenance (phase 2: weeks 25–48), and observation (phase 3: weeks 49–72) (Figure 1).

Survey data were available for the EQ‐5D‐3L Index and VR‐12 in 877 and 875 participants, respectively, at baseline; 748 and 759 at 24 weeks; 761 and 750 at 48 weeks; and 691 and 692 at 72 weeks. Participants with gout and serum urate level >6.8 mg/dL were randomly assigned to allopurinol or febuxostat. For those not taking allopurinol at enrollment, agents were initiated at daily doses of 100 mg for allopurinol and 40 mg for febuxostat with doses gradually escalated to achieve a serum urate level <6.0 mg/dL (<5.0mg/dL if tophi were present) or until the maximal allowable urate‐lowering therapy dose was reached. Participants taking allopurinol ≤300 mg daily before the trial were eligible and continued taking their pretrial (blinded) allopurinol dose if relevant and randomly assigned to that arm, with dose titration delayed for those receiving 200 mg (starting at week 6) or 300 mg (starting at week 9). The dose titration schedule^13^ during phase 1 followed the 2012 ACR gout management guidelines,^14^, ^15^ with maximum daily doses of 800 mg of allopurinol and 120 mg of febuxostat. The maximum allowable daily febuxostat dose was reduced from 120 mg to 80 mg during the study, in February 2019, at the request of the US Food and Drug Administration following a modification of febuxostat labeling to include a black box warning detailing cardiovascular safety concerns.^16^ All participants received guideline‐directed anti‐inflammatory prophylaxis per 2012 ACR guidelines^15^ with colchicine, naproxen 250 mg twice daily, or prednisone ≤10 mg daily per investigator discretion that extended through the entirety of phase 1. Additional prophylaxis was allowed during phase 2 with discontinuation mandatory before the end of this phase (by the end of week 48).

Adherence to urate‐lowering therapy was assessed throughout the trial using participant‐maintained diaries. Adherence data were available for 83% of participants, with compliance (defined as taking the assigned study urate‐lowering therapy ≥80% of days) reported in 84.2%.^17^ The trial indicated that allopurinol was noninferior to febuxostat in controlling flares, with similar achievement of serum urate goals (80% overall achieved serum urate goal) and no significant differences in serious adverse events.^13^

### HRQoL questionnaires

Two questionnaires, the VR‐12 and EQ‐5D‐3L, were administered to STOP Gout participants at baseline and at 24, 48, and 72 weeks. The VR‐12 was developed and modified from the original RAND version of the 12‐Item Health Survey version 1.0 (also known as the 12‐item Short Form [SF‐12]).^18^, ^19^, ^20^, ^21^, ^22^, ^23^ Similar to the SF‐12, VR‐12 results are reported in two scores: a physical component score (PCS) and mental component score (MCS), each standardized to the VA population mean ± SD of 50 ± 10 with higher scores reflecting better HRQoL. Derived from the VR‐12, the Veterans RAND 6‐Dimension (VR‐6D) survey is a single HRQoL utility index with a range of 0 to 1 with 0 representing death and 1 perfect health.^24^ Similarly, the EQ‐5D‐3L includes five items, each with three possible responses, addressing the HRQoL domains of mobility, self‐care, activity, pain, and mental health. The EQ‐5D‐3L with US‐based tariffs produces an overall index score with range 0 to 1 with 0 representing death and 1 representing perfect health. In contrast, higher scores in individual EQ‐5D‐3L domains (range 1–3) reflect worse HRQoL. To facilitate data interpretation, individual EQ‐5D‐3L domain scores were reversed such that higher scores reported herein denote better HRQoL.

### Analyses

Changes in VR‐6D and EQ‐5D‐3L index scores from baseline to 72 weeks among all participants (allopurinol and febuxostat arms pooled) served as the co‐primary endpoints for this post hoc analysis. Descriptive statistics were used to compare participant characteristics by urate‐lowering therapy treatment assignment (ie, allopurinol vs febuxostat) as previously reported^13^ in addition to comparing characteristics of those completing the 72‐week study and those dropping out at earlier time points. In initial cross‐sectional analyses, factors associated with baseline HRQoL in participants with gout were assessed using univariable linear regression. All variables with *P* < 0.1 in univariable analyses were then included in a multivariable linear regression model. In these analyses, beta coefficients (β) and 95% confidence intervals (CIs) were generated and multiplied by a factor of 100 to facilitate interpretation. Variables examined included demographics (age, sex, race, ethnicity, and urban/rural residence), urate‐lowering therapy assignment, gout duration, presence of tophi, prior allopurinol receipt, high‐sensitivity C‐reactive protein (hs‐CRP) concentration (mg/L), serum urate (mg/dL), presence of Stage 3 CKD, overall comorbidity burden as assessed by the Rheumatic Disease Comorbidity Index (RDCI),^25^ diuretic use, body mass index (kg/m^2^), and regular alcohol use. The presence of component conditions making up the RDCI were identified using diagnostic codes from linked electronic health record data contained within the VA Corporate Data Warehouse. Both race and ethnicity were self‐reported by participants in response to open‐ended questions posed by study personnel. Non‐VA study participants with missing RDCI values (n = 62) were excluded from this analysis in addition to the longitudinal analyses detailed below, leaving 878 total participants.

Changes in the VR‐6D survey, EQ‐5D‐3L index, and component scores between baseline and 72‐week values were examined using paired *t*‐tests. Radar plots^26^ were used to graphically demonstrate changes in individual EQ‐5D‐3L components. Participants with missing HRQoL values from either of the two relevant time points were excluded from analyses (Figure 1). Among those with baseline EQ‐5D‐3L data (n = 877), 14.7% (n = 129) were missing data from 24 weeks, 13.2% (n = 116) from 48 weeks, and 21.2% (n = 186) from 72 weeks. For those with baseline VR‐12 data (n = 875), 13.3% were missing data from 24 and 48 weeks (n = 116 for both) and 20.9% (n = 183) were missing data from 72 weeks. To further explore whether participant attrition impacted changes in HRQoL observed over follow‐up, we performed an additional analysis restricted to individuals with complete HRQoL data available at all study time points. A standardized mean gain (SMG) was generated to quantify the magnitude of change observed for each measure over follow‐up by calculating the mean change between baseline and a later period divided by the SD at baseline. Values of 0.20 were indicative of a small effect, 0.50 a medium effect, and >0.80 a large effect.^27^ General estimating equations (GEEs) were used to examine associations of participant factors with change in HRQoL from baseline to 24, 48, and 72 weeks. In addition to the same covariates as detailed above in cross‐sectional analyses, additional variables examined in GEE models included baseline HRQoL values, flare counts during the preceding study phase, the achievement of serum urate goal in the previous phase as determined by blood draws taken at the completion of the phase, and anti‐inflammatory prophylaxis use (colchicine vs other).

### Ethical considerations

The STOP Gout study was approved by the VA Central Institutional Review Board, and all participants provided written informed consent before enrollment. The STOP Gout Steering Committee provided approval for the conduct of this post hoc analysis.

## RESULTS

Characteristics of the 878 study participants included in the analysis are summarized in Table 1. The cohort was primarily male (98.9%), had self‐reported White race (67.4%), had a mean age of 62.4 years, had a mean serum urate of 8.53 mg/dL, and had a mean disease duration of 10 years at enrollment. Approximately one‐third (37.8%) of participants had CKD Stage 3, and 16.6% had tophi. Approximately one‐third (37%) of participants had previously used allopurinol. Baseline HRQoL measures are shown in Table 2. Overall, participants completing the study were similar to those dropping out at earlier time points except that noncompleters had a worse baseline EQ‐5D‐3L score (0.66 vs 0.71) and were more likely to live in an urban area (Supplementary Table S1).

**Table 1:** Baseline characteristics of study participants (N = 878)*

Baseline characteristics	Data
Demographics	
Age, mean (SD), y	62.4 (12.2)
Male, %	98.9
Race, %	
White/Caucasian	67.4
Black/African American	22.2
Other	10.4
Hispanic ethnicity, %	5.0
Urban residence, %	73.3
Gout‐related factors	
Serum urate level, mean (SD), mg/dL	8.53 (1.37)
C‐reactive protein, mean (SD), mg/L	8.98 (17.11)
Gout duration, mean (SD), y	10.0 (11.1)
Prior allopurinol use, %	37.0
Presence of tophi, %	16.6
Treatment assigned, %	
Allopurinol	50.0
Febuxostat	50.0
Comorbidities	
Chronic kidney disease, Stage 3, %	37.8
Diuretic use, %	43.5
RDCI	2.2 (1.57)
BMI (kg/m^2^), %	
<25 (healthy)	5.3
25 ≤ BMI < 30 (overweight)	27.6
30 ≤ BMI < 35 (obese)	31.7
≥35 (morbidly obese)	35.4
Alcohol use, %	54.5

* “Other” race combines Asian, American Indian or Alaska Native, Native Hawaiian or other Pacific Islander, and “none of the above.” BMI, body mass index; RDCI, Rheumatic Disease Comorbidity Index.

**Table 2:** VR‐12 (derived VR‐6D) and EQ‐5D‐3L scores in participants receiving treat‐to‐target urate‐lowering therapy at baseline, 24, 48, and 72 weeks*

	Initial	24 weeks	48 weeks	72 weeks
Overall HRQoL measures				
VR‐6D, mean (SD)	0.66 (0.12)	0.70 (0.12)	0.70 (0.12)	0.70 (0.12)
Change from initial, mean (SD)	–	0.04 (0.10)	0.03 (0.10)	0.04 (0.10)
*P* value	–	<0.001	<0.001	<0.001
Effect size, SMG	–	0.31	0.28	0.30
n	875	759	750	692
EQ‐5D‐3L index, mean (SD)	0.70 (0.22)	0.75 (0.21)	0.75 (0.22)	0.74 (0.22)
Change from initial, mean (SD)	–	0.05 (0.18)	0.04 (0.19)	0.03 (0.19)
*P* value	–	<0.001	<0.001	<0.001
Effect size, SMG	–	0.22	0.18	0.12
n	877	748	761	692
VR‐12 component scores				
VR‐12 Physical (PCS), mean (SD)	36.8 (10.9)	40.7 (11.5)	40.6 (11.5)	40.9 (11.5)
Change from initial, mean (SD)	–	3.9 (9.8)	3.6 (9.9)	3.7 (10.4)
*P* value	–	<0.001	<0.001	<0.001
Effect size, SMG	–	0.35	0.32	0.33
VR‐12 Mental (MCS), mean (SD)	51.3 (11.5)	51.9 (11.0)	52.1 (10.7)	52.0 (10.8)
Change from initial, mean (SD)	–	0.4 (9.7)	0.4 (10.3)	0.4 (10.7)
*P* value	–	0.28	0.35	0.38
Effect size, SMG	–	0.03	0.03	0.03
EQ‐5D‐3L dimension scores				
Mobility, mean (SD)	2.45 (0.50)	2.58 (0.50)	2.57 (0.50)	2.54 (0.51)
Change from initial, mean (SD)	–	0.13 (0.53)	0.12 (0.53)	0.09 (0.52)
*P* value	–	<0.001	<0.001	<0.001
Effect size, SMG	–	0.26	0.24	0.18
Self‐care, mean (SD)	2.87 (0.34)	2.88 (0.34)	2.87 (0.35)	2.86 (0.38)
Change from initial, mean (SD)	–	0.00 (0.35)	−0.01 (0.34)	−0.02 (0.36)
*P* value	–	0.75	0.52	0.10
Effect size, SMG	–	0.01	−0.02	−0.07
Daily activities, mean (SD)	2.55 (0.55)	2.64 (0.51)	2.61 (0.54)	2.59 (0.53)
Change from initial, mean (SD)	–	0.08 (0.55)	0.06 (0.59)	0.04 (0.59)
*P* value	–	<0.001	0.009	0.30
Effect size, SMG	–	0.15	0.10	0.04
Pain, mean (SD)	2.19 (0.58)	2.39 (0.58)	2.37 (0.58)	2.36 (0.58)
Change from initial, mean (SD)	–	0.18 (0.64)	0.16 (0.63)	0.14 (0.62)
*P* value	–	<0.001	<0.001	<0.001
Effect size, SMG	–	0.32	0.28	0.25
Mood, mean (SD)	2.67 (0.53)	2.68 (0.51)	2.68 (0.52)	2.64 (0.52)
Change from initial, mean (SD)	–	−0.02 (0.48)	−0.02 (0.49)	−0.03 (0.49)
*P* value	–	0.33	0.21	0.06
Effect size, SMG	–	−0.03	−0.04	−0.07

* All changes are calculated using only patients who had measurements at both points. EQ‐5D‐3L, EuroQol 5‐Dimension 3‐Level; HRQoL, health‐related quality of life; MCS, mental component score; PCS, physical component score; SMG, standard mean gain; VR‐6D, Veterans RAND 6‐Dimension; VR‐12, Veterans RAND 12‐item.

**Figure 2:**
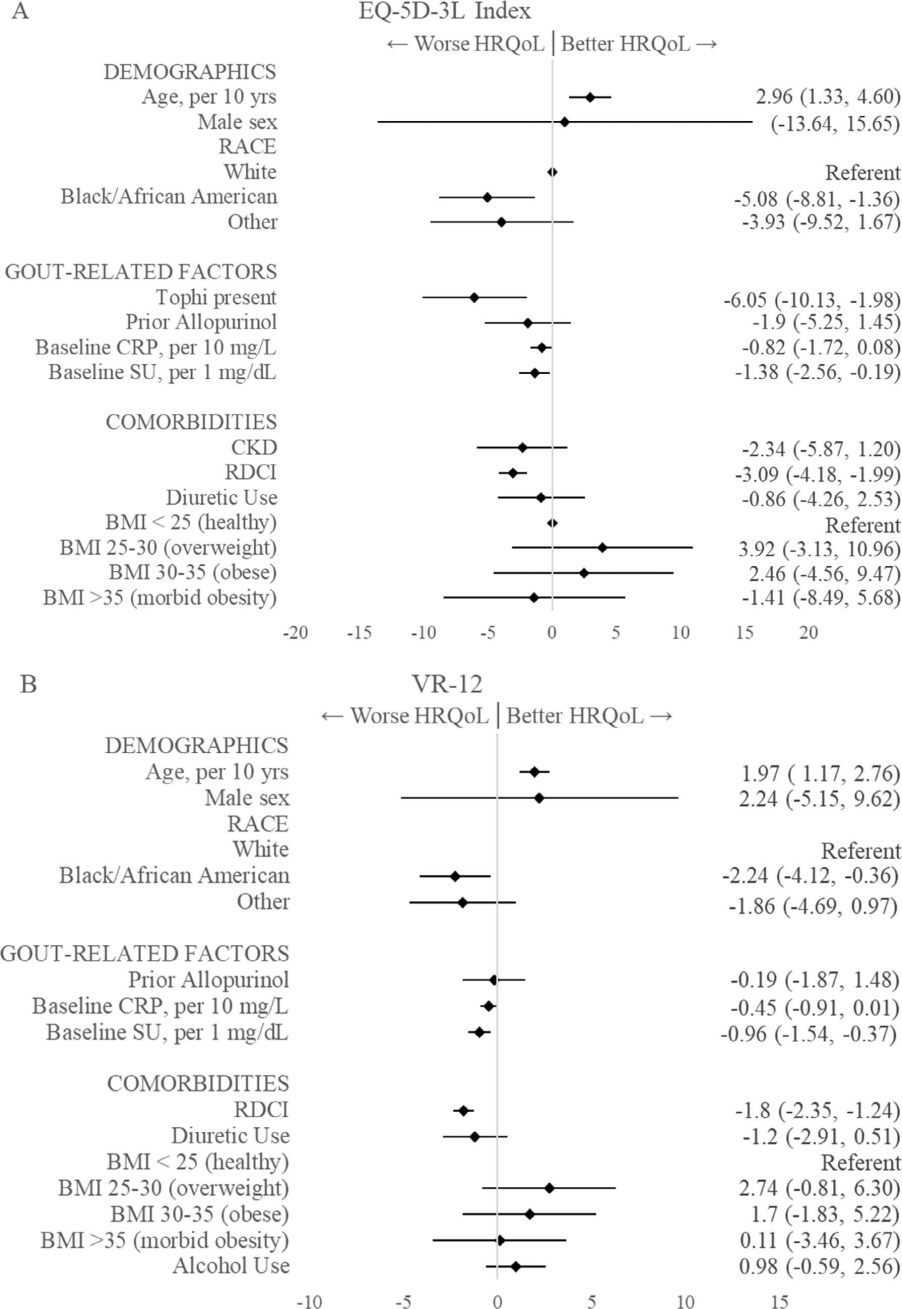
Multivariable linear regression of participant factors associated with baseline HRQoL in STOP Gout participants before initiating treat‐to‐target urate‐lowering therapy. Beta coefficients and 95% confidence interval shown for (A) EQ‐5D‐3L Index and (B) VR‐6D. Only factors with *P* value <0.1 in univariable analysis were included. EQ‐5D‐3L Index and VR‐6D scores were multiplied by 100. Negative scores are associated with worse baseline HRQoL. “Other” race combines Asian, American Indian or Alaska Native, Native Hawaiian or other Pacific Islander, and “none of the above.” BMI, body mass index; CKD, chronic kidney disease; CRP, C‐reactive protein; EQ‐5D‐3L, EuroQol 5‐Dimension 3‐Level; HRQoL, health‐related quality of life; RDCI, Rheumatic Disease Comorbidity Index; SU, serum urate; VR‐12, Veterans RAND 12‐item.

Univariable associations of participant characteristics with baseline/enrollment HRQoL are summarized in Supplementary Table S2. In multivariable models, factors independently associated with worse enrollment/baseline HRQoL included younger age, Black/African American race (vs White), presence of tophi (EQ‐5D‐3L), higher baseline serum urate, and higher RDCI score (Figure 2).

**Figure 3:**
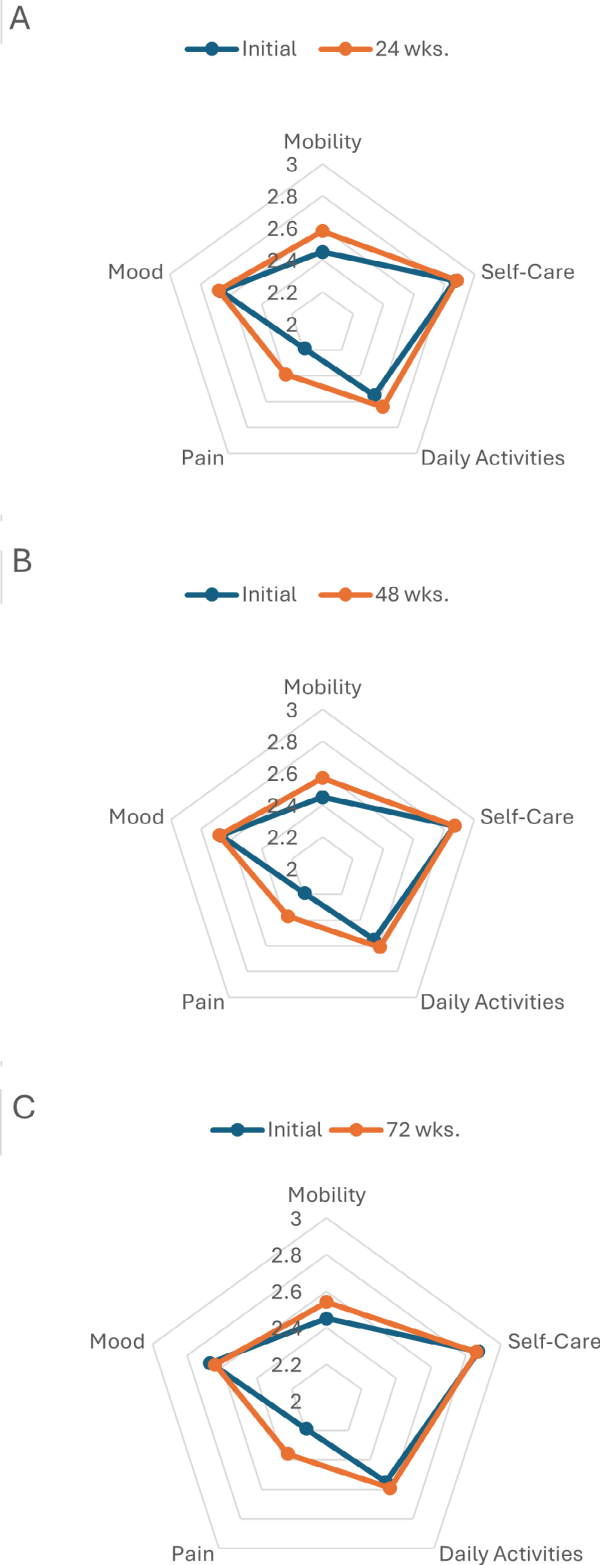
Radar plots depicting change in the five dimensions (mobility, self‐care, daily activities, pain, and mood) of the EuroQol 5‐Dimension 3‐Level from baseline to (A) 24 weeks, (B) 48 weeks, and (C) 72 weeks. Data were available for 877 participants at baseline, 748 at 24 weeks, 761 at 48 weeks, and 692 at 72 weeks.

Mean ± SD HRQoL scores at baseline 24, 48, and 72 weeks are shown in Table 2. The mean overall VR‐6D score at enrollment was 0.66 ± 0.12 with a baseline PCS of 36.8 ± 10.9 and an MCS of 51.3 ± 11.5. There were improvements in both the VR‐6D and PCS by 24 weeks, which were sustained and significant at 72 weeks of follow‐up (SMG 0.30 and 0.33 vs baseline, respectively, *P* < 0.001 for both). There were no significant changes in VR‐12 MCS observed. Similar improvements in the EQ‐5D‐3L index score were observed, albeit of slightly lower magnitude (SMG 0.12, *P* < 0.001 at 72 weeks). Of the EQ‐5D‐3L dimensions assessed, only mobility (SMG 0.18) and pain (SMG 0.25) demonstrated evidence of improvement at 72 weeks (*P* < 0.001 for both) (Figure 3). There were no differences in the dimensions of self‐care, activities, or mood at 72 weeks. In an additional analysis limited to participants with available HRQoL data from all study time points, changes in HRQoL over follow‐up were nearly identical to those from our primary analysis that included data from all available individuals at each time point (Supplementary Table S3).

Results from GEE models examining factors associated with VR‐6D and EQ‐5D‐3L changes at 24, 48, and 72 weeks from baseline are shown in Table 3. Improvements in both VR‐6D and EQ‐5D‐3L index scores were associated with lower baseline hs‐CRP (β −0.59 [95% CI −1.12 to −0.06] and −1.20 [95% CI −2.29 to −0.11], respectively, per 10 g/L) and lower comorbidity burden as assessed by the RDCI (β −1.26 [95% CI −1.67 to −0.85] and −1.77 [95% CI −2.49 to −1.06], respectively). Consistent with similar outcomes noted in the parent trial,^13^ there was no difference in HRQoL based on whether participants were randomized to allopurinol or febuxostat. Contrary to our a priori hypothesis, serum urate goal achievement at the end of the previous phase (observed in 83% at 24 weeks and 78% at 48 weeks) was not significantly associated with changes in HRQoL (Table 3). Although not achieving statistical significance, there were trends suggesting that those experiencing more frequent flares (at least two vs none; observed in 24% in phase 1 and 16% in phase 2) during the prior study phase were less likely to experience improvements in VR‐6D (β −1.20; 95% CI −3.09 to 0.70) or EQ‐5D‐3L (β −0.86; 95% CI −1.86 to 0.13).

**Table 3:** General estimating equation models examining factors associated with EQ‐5D‐3L and VR‐12 changes at 24, 48, and 72 weeks versus baseline*

	EQ‐5D‐3L index		VR‐6D	
	β (95% CI)	*P* value	Β (95% CI)	*P* value
EQ‐5D index baseline	−0.43 (−0.49 to −0.38)	<0.001	–	–
VR‐12 baseline	–	–	−0.37 (−0.42 to −0.31)	<0.001
Demographics				
Age per 10 y	−0.41 (−1.48 to 0.65)	0.45	0.09 (−0.49 to 0.66)	0.77
Male sex	−0.28 (−5.95 to 5.39)	0.92	1.50 (−1.85 to 4.86)	0.38
Race				
White	Ref	Ref.	Ref	Ref.
Black/African American	−0.54 (−3.37 to 2.28)	0.71	0.33 (−1.36, 2.02)	0.70
Other	0.64 (−3.65 to 4.94)	0.77	0.80 (−1.71 to 3.31)	0.53
Hispanic ethnicity	1.62 (−2.94 to 6.19)	0.49	−0.28 (−2.96 to 2.40)	0.84
Urban residence	−1.87 (−4.12 to 0.39)	0.10	−0.91 (−2.28 to 0.45)	0.19
Gout‐related factors				
Gout duration, per 10 y	−0.65 (−1.80 to 0.50)	0.27	−0.51 (−1.12 to 0.11)	0.11
Tophi present	1.88 (−0.92 to 4.67)	0.19	1.40 (−0.24 to 3.03)	0.09
Prior allopurinol use	−1.08 (−3.21 to 1.06)	0.32	0.07 (−1.21 to 1.34)	0.92
Baseline serum urate per 1 mg/dL	−0.59 (−1.36, 0.18)	0.13	0.07 (−0.37 to 0.50)	0.77
Treatment assigned				
Allopurinol	Ref	Ref.	Ref	Ref.
Febuxostat	0.59 (−1.42 to 2.59)	0.57	0.17 (−1.01 to 1.36)	0.77
Prophylaxis with colchicine	−1.58 (−4.97 to 1.81)	0.36	−0.89 (−2.83 to 1.05)	0.37
Flares in preceding phase				
0 flares	Ref	Ref.	Ref	Ref.
1 flare	−0.33 (−2.13 to 1.47)	0.72	0.16 (−0.75 to 1.08)	0.73
≥2 flares	−1.20 (−3.09 to 0.70)	0.22	−0.86 (−1.86 to 0.13)	0.09
Serum urate level at goal (end of prior phase)	0.52 (−1.35 to 2.38)	0.59	0.44 (−0.62 to 1.51)	0.41
Baseline hs‐CRP per 10 mg/L	−1.20 (−2.29 to −0.11)	0.03	−0.59 (−1.12 to 0.06)	0.03
Comorbidities				
CKD Stage 3 (vs stage 1/2)	0.30 (−2.01 to 2.60)	0.80	0.23 (−1.09 to 1.54)	0.74
RDCI	−1.77 (−2.49 to −1.06)	<0.001	−1.26 (−1.67 to −0.85)	<0.001
BMI (kg/m^2^)				
<25 (healthy)	Ref	Ref.	Ref	Ref.
25–30 (overweight)	0.15 (−3.48 to 3.79)	0.94	0.17 (−2.35 to 2.70)	0.89
30–35 (obese)	−0.33 (−4.06 to 3.40)	0.86	0.82 (−1.75 to 3.39)	0.53
>35 (morbid obesity)	−2.43 (−6.24 to 1.37)	0.21	−0.55 (−3.16 to 2.05)	0.68
Alcohol use	−1.22 (−3.27 to 0.83)	0.24	−0.81 (−2.03 to 0.40)	0.19

* “Other” race combines Asian, American Indian or Alaska Native, Native Hawaiian or other Pacific Islander, and “none of the above.” BMI, body mass index; CI, confidence interval; CKD, chronic kidney disease; EQ‐5D‐3L, EuroQol 5‐Dimension 3‐Level; hs‐CRP, high‐sensitivity C‐reactive protein; RDCI, Rheumatic Disease Comorbidity Index; Ref, reference; VR‐6D, Veterans RAND 6‐Dimension;VR‐12, Veterans RAND 12‐item.

## DISCUSSION

In this post hoc analysis using data from the STOP Gout trial,^13^ participants receiving treat‐to‐target urate‐lowering therapy demonstrated significant improvements in HRQoL over the 72‐week study period. Improvements observed in both the VR‐6D and EQ‐5D‐3L index scores were evident as early as 24 weeks and corresponded to small‐to‐moderate effect sizes. These improvements in HRQoL were driven largely by gains in the PCS of the VR‐12 as well as in the pain and mobility components of the EQ‐5D‐3L. Although representing only small‐to‐moderate effects, changes observed in the VR‐12 PCS approach or surpass the minimal important difference in this measure estimated for other musculoskeletal conditions.^28^, ^29^, ^30^ These findings provide further support for treat‐to‐target urate‐lowering therapy in gout management. In contrast, treat‐to‐target urate‐lowering therapy was not accompanied by meaningful improvements in the MCS of the VR‐12 or other HRQoL domains of the EQ‐5D‐3L (daily activities, mood, and self‐care).

Results of this analysis complement and expand on those from other reports examining the impact of treat‐to‐target urate‐lowering therapy on HRQoL.^10^, ^11^, ^12^ A nurse‐led trial in the United Kingdom showed improvements in HRQoL among participants with gout randomized to receive treat‐to‐target urate‐lowering therapy,^12^ improvements that were evident as early as 1 year following treatment initiation. In contrast, a parallel group of participants receiving only “usual gout care” demonstrated no meaningful changes in HRQoL over the same follow‐up period. As in our analysis, improvements gained with treat‐to‐target urate‐lowering therapy in the UK study were similar in magnitude and limited to the PCS (SMG 0.38) and did not extend to the MCS (measured in the UK study using the more comprehensive 36‐item Short Form [SF‐36] instrument). The earlier 6‐month assessment available from the STOP Gout study suggests that gains in HRQoL are made sooner in the course of treat‐to‐target urate‐lowering therapy than 1 year. The potential for earlier benefit in HRQoL is further supported by findings from the NOR‐Gout trial, in which improvements in work participation and activity were evident within 3 months of treat‐to‐target urate‐lowering therapy initiation.^10^ Sustained benefit, observed in both the STOP Gout and NOR‐Gout studies over 72 weeks and 2 years of follow‐up, respectively, indicates that HRQoL improvements derived from treat‐to‐target urate‐lowering therapy are likely independent of anti‐inflammatory prophylaxis used earlier in treatment. Taken together, the results of these studies support both early and sustained benefit from treat‐to‐target urate‐lowering therapy on HRQoL using first‐line therapy (allopurinol or febuxostat) and the importance of treat‐to‐target urate‐lowering therapy in improving patient‐centered outcomes in gout.

In addition to demonstrating benefits accompanying treat‐to‐target urate‐lowering therapy administration, data from the STOP Gout trial also provided an opportunity to identify factors associated with baseline HRQoL in participants with gout before initiating treat‐to‐target urate‐lowering therapy as well as characteristics associated with improvements gained with best‐practice management. In this post hoc analysis, factors independently associated with worse HRQoL in participants at the time of trial enrollment included younger age, Black/African American race (vs White), presence of tophi, higher baseline serum urate, and a higher comorbidity burden. These results align with those from separate investigations demonstrating associations of non‐White race, gout severity, and gout‐related comorbidities with reductions in HRQoL.^2^, ^4^, ^31^, ^32^, ^33^, ^34^, ^35^, ^36^ Previously, results from the STOP Gout study also showed that younger participants and those self‐reporting non‐White race were also less likely to achieve serum urate goals^17^ whereas younger patients were more likely to experience arthritis flares complicating the course of treat‐to‐target urate‐lowering therapy.^37^ Alternatively, a younger age of gout onset has been proposed as an indicator of a more severe disease phenotype. In addition to being more recalcitrant to urate‐lowering therapy, earlier‐onset gout has been associated with higher pretreatment serum urate level and a greater flare burden.^38^ Associations of younger age with worse outcomes in gout contrasts with observations from the general population wherein HRQoL generally decreases with advancing age.^39^ Although the reasons underpinning these different results across populations remain unknown, one possibility is that age‐related differences in genetic factors influencing both disease susceptibility and outcome, drug metabolism, or changes in xanthine oxidase expression render younger patients less likely to benefit from treatment.

Beyond ascertaining gout‐related determinants of HRQoL at enrollment, the availability of longitudinal trial data enabled the identification of factors associated with HRQoL improvements accompanying highly effective urate‐lowering therapy. To date, there has been minimal evaluation of the factors associated with change in HRQoL produced within a treat‐to‐target framework. The results of the GEE models showed that improvements in HRQoL, whether measured by the VR‐6D or EQ‐5D‐3L, were associated with a lower baseline comorbidity burden (lower RDCI score) and lower baseline levels of systemic inflammation as assessed by hs‐CRP. The associations of comorbidity noted herein parallel findings from the NOR‐Gout trial, demonstrating that fewer comorbidities were associated with greater improvements in work participation in the wake of treat‐to‐target gout management.^10^ The reduced treatment response apparent among those with greater comorbidity may simply reflect QoL deficits that are independent of gout and the gout management strategies deployed.

In contrast to our a priori hypotheses, we did not detect associations of gout flares or serum urate goal achievement with HRQoL change. Although this study afforded a large sample size of participants receiving treat‐to‐target urate‐lowering therapy, we had limited study power to address these post hoc questions, particularly as approximately 80% achieved the serum urate goal during the parent trial and >90% achieved serum urate concentrations <6.8 mg/dL.^13^ Moreover, we were unable to assess flare severity in this analysis (eg, number of joints involved or pain intensity), which could be more closely related to changes in HRQoL than simple flare occurrence or count. Consistent with the noninferiority of allopurinol to febuxostat for the primary and secondary outcomes of flare reduction and serum urate goal achievement previously reported,^13^ urate‐lowering therapy assignment did not meaningfully impact HRQoL change.

There are limitations to this study. Although the US veteran population examined in this analysis demonstrates similar characteristics as those reported in other gout trials,^40^, ^41^, ^42^ the results herein may not be universally generalizable. This issue might be most relevant among women, who made up <2% of STOP Gout participants.^13^ Given similar findings of efficacy in the parent trial by treatment assignment, data from the original allopurinol and febuxostat arms were pooled for these analyses and all participants were administered urate‐lowering therapy following a treat‐to‐target protocol. In the absence of an untreated or “usual care” comparator population, it is possible that improvements observed in HRQoL observed could attribute to other components of trial participation beyond urate‐lowering therapy. Although the STOP Gout trial served as the source of data for this analysis, the post hoc study reported herein does not benefit from the initial randomization and has many of the limitations inherent to an observational study, including the possibility of participation bias or changes observed in HRQoL representing “regression to the mean.”^43^ Although impacting only a limited proportion of participants, it is also possible that study attrition could have biased findings from this analysis because individuals experiencing fewer benefits from the urate‐lowering therapy intervention, including HRQoL improvements, may have also been more likely to have withdrawn from the study. This possibility is highlighted by differences observed in baseline EQ‐5D‐3L scores and residence of participants completing the study through 72 weeks and those dropping out. However, an analysis of participants with HRQoL data available at all time points closely mirrored that of the overall study population, suggesting that attrition had minimal impact on these results.

In contrast to more comprehensive assessments used in other gout studies such as the SF‐36,^11^, ^12^ the VR‐12 and EQ‐5D‐3L instruments are brief, both requiring one‐third of the time or less to complete. Although these generic health surveys may provide for greater study efficiency, the lower number of options for each item can result in a ceiling effect, defined as the inability of a questionnaire to capture increases in the measured attribute if participants already score at or near the maximum value.^39^ Given the generic nature of the questionnaires used, it is possible that the use of a gout‐specific instrument such as the Gout Impact Scale could have shown greater gains in HRQoL accompanying treat‐to‐target management.^44^ However, we would anticipate that the generic nature of the questionnaires and any ceiling effect would both bias results toward the null, suggesting that our findings represent conservative estimates of HRQoL changes attending treat‐to‐target urate‐lowering therapy.

In conclusion, by leveraging data available from a large comparative trial of allopurinol and febuxostat, this analysis demonstrates that treat‐to‐target urate‐lowering therapy is accompanied by significant improvements in HRQoL. These improvements are most prominent in the domains of physical function, pain, and mobility; are evident relatively early in the course of treat‐to‐target therapy; and are sustained over longer periods of follow‐up. Moreover, gout subgroups characterized by lower comorbidity burden and lower levels of systemic inflammation appear to experience the greatest improvements in HRQoL with treat‐to‐target urate‐lowering therapy.

## AUTHOR CONTRIBUTIONS

All authors contributed to at least one of the following manuscript preparation roles: conceptualization AND/OR methodology, software, investigation, formal analysis, data curation, visualization, and validation AND drafting or reviewing/editing the final draft. As corresponding author, Dr Mikuls confirms that all authors have provided the final approval of the version to be published and takes responsibility for the affirmations regarding article submission (eg, not under consideration by another journal), the integrity of the data presented, and the statements regarding compliance with institutional review board/Declaration of Helsinki requirements.

## Supporting information


**Disclosure Form**:

**Supplemental Table 1** Baseline characteristics of participants with data available for both HRQoL surveys (VR and EQ‐5D‐3L) at baseline and 72 weeks (Completers) vs participants missing data for either survey at week 72 (Non‐Completers).
**Supplemental Table 2:** Univariable associations of participant factors with baseline health‐related quality of life. EQ‐5D‐3L Index and VR‐6D results multiplied by 100. Negative scores are associated with worse baseline HRQoL.
**Supplemental Table 3:** Veterans Rand (VR)‐12 (derived VR‐6D) and EurolQol‐5‐Dimension‐3‐Level (EQ‐5D‐3L) scores in participants with data available at all time points receiving treat‐to‐target urate‐lowering therapy at baseline, 24‐, 48‐ and 72‐wks; mean values shown (SD).
