# Supplementary material for: Improvements in Health‐Related Quality of Life With Treat‐to‐Target Urate‐Lowering Therapy in Gout: A Post Hoc Analysis of a Randomized Multicenter Trial
Source: Arthritis Care Res (Hoboken). 2025 Nov 17;78(2):259–69. doi: 10.1002/acr.25618 (PMC12919703; doi:10.1002/acr.25618)
Supplement: Supplementary file 2 — Supplemental Table 1 Baseline characteristics of participants with data available for both HRQoL surveys (VR and EQ‐5D‐3L) at baseline and 72 weeks (Completers) vs participants missing data for either survey at week 72 (Non‐Completers). Supplemental Table 2: Univariable associations of participant factors with baseline health‐related quality of life. EQ‐5D‐3L Index and VR‐6D results multiplied by 100. Negative scores are associated with worse baseline HRQoL. Supplemental Table 3: Veterans Rand (VR)‐12 (derived VR‐6D) and EurolQol‐5‐Dimension‐3‐Level (EQ‐5D‐3L) scores in participants with data available at all time points receiving treat‐to‐target urate‐lowering therapy at baseline, 24‐, 48‐ and 72‐wks; mean values shown (SD). [file ACR-78-259-s001.docx]

**Supplemental Table 1:** Baseline characteristics of participants with data available for both HRQoL surveys (VR and EQ-5D-3L) at baseline and 72 weeks (Completers) vs participants missing data for either survey at week 72 (Non-Completers).

| **BASELINE CHARACTERISTICS** | **Completers** | **Non-Completers** | **P-value** |
| --- | --- | --- | --- |
|  | N=691 | N=186 |  |
| Baseline VR-6D Score | 0.67 (0.12) | 0.64 (0.11) | 0.013 |
| Baseline EQ-5D-3L Score | 0.71 (0.21) | 0.66 (0.25) | 0.003 |
| **DEMOGRAPHICS** | | | |
| Age, years, mean (SD) | 62.7 (11.8) | 61.2 (13.5) | 0.16 |
| Male, % | 99 | 99 | 0.70 |
| Race, %  White/Caucasian  Black/African American  Other | 69  21  10 | 61  28  11 | 0.09 |
| Hispanic ethnicity, % | 6 | 3 | 0.10 |
| Urban residence, % | 72 | 80 | 0.029 |
| **GOUT-RELATED FACTORS** | | | |
| Serum Urate mg/dL, mean (SD) | 8.5 (1.4) | 8.5 (1.3) | 0.55 |
| C-reactive protein mg/L, mean (SD) | 8.7 (16.2) | 9.9 (20.1) | 0.55 |
| Gout duration, years, mean (SD) | 9.7 (10.9) | 10.9 (11.6) | 0.18 |
| Prior Allopurinol use, % | 38 | 32 | 0.09 |
| Presence of tophi, % | 16 | 20 | 0.12 |
| Treatment Assigned, %  Allopurinol  Febuxostat | 49  51 | 52  48 | 0.52 |
| **CORMORBIDITIES** | | | |
| Chronic kidney disease, stage 3, % | 38 | 38 | 0.89 |
| Diuretic use, % | 44 | 42 | 0.71 |
| RDCI | 2.2 (1.6) | 2.1 (1.6) | 0.58 |
| Body mass index (BMI), kg/m^2^, %  < 25 (healthy)  25 ≤ BMI < 30 (overweight)  30 ≤ BMI < 35 (obese)  ≥ 35 (morbidly obese) | 5  28  30  37 | 5  25  38  31 | 0.20 |
| Alcohol use, % | 54 | 55 | 0.86 |

‘Other’ race combines Asian, American Indian or Alaska Native, Native Hawaiian or other Pacific Islander and ‘none of the above’; RDCI, Rheumatic Disease Comorbidity Index

**Supplemental Table 2:** Univariable associations of participant factors with baseline health-related quality of life. EQ-5D-3L Index and VR-6D results multiplied by 100. Negative scores are associated with worse baseline HRQoL.

|  | **EQ-5D-3L Index** | | **VR-6D** | |
| --- | --- | --- | --- | --- |
|  | **Beta coefficient (95% CI)** | **P-value** | **Beta Coefficient (95% CI)** | **P-value** |
| **DEMOGRAPHICS** | | | | |
| Age, per 10 yrs | 1.28 (0.79, 2.49) | 0.04 | 1.45 (0.83, 2.07) | <0.001 |
| Male sex | 0.62 (-13.26, 14.50) | 0.93 | 2.53 (-4.70, 9.76) | 0.49 |
| Race  White  Black/African American  Other | Ref.  -4.88 (-8.47, -1.29)  -3.28 (-8.20, 1.64) | Ref.  0.01  0.19 | Ref.  -2.58 (-4.45, -0.71)  -2.98 (-5.54, -0.42) | Ref.  0.01  0.02 |
| Hispanic ethnicity | -1.16 (-7.91, 5.59) | 0.74 | -1.47 (-5.00, 2.05) | 0.41 |
| Urban Residence | -2.38 (-5.71, 0.95) | 0.16 | -1.29 (-3.02, 0.45) | 0.15 |
| **GOUT-RELATED FACTORS** | | | | |
| Febuxostat (vs. Allopurinol) | 1.15 (-1.80, 4.09) | 0.45 | 0.37 (-1.16, 1.91) | 0.63 |
| Gout Duration, per 10 yrs | -0.71 (-2.04, 0.62) | 0.30 | 0.08 (-0.62, 0.77) | 0.83 |
| Tophi present | -5.24 (-9.18, -1.30) | 0.01 | -1.02 (-3.08, 1.05) | 0.33 |
| Prior Allopurinol | -3.30 (-6.35, -0.26) | 0.03 | -1.54 (-3.13, 0.04) | 0.06 |
| Baseline CRP, per 10 mg/L | -1.81 (-2.68, -0.94) | <0.001 | -0.84 (-1.30, -0.39) | <0.001 |
| Base SU, per 1 mg/dL | -2.46 (-3.52, -1.39) | <0.001 | -1.54 (-2.09, -0.99) | <0.001 |
| **COMORBIDITIES** | | | | |
| CKD | -3.55 (-6.58, -0.51) | 0.02 | -0.34 (-1.92, 1.25) | 0.68 |
| RDCI | -2.99 (-3.91, -2.08) | <0.001 | -1.51 (-1.99, -1.03) | <0.001 |
| Diuretic Use | -3.27 (-6.42, -0.11) | 0.04 | -2.26 (-3.88, -0.65) | 0.01 |
| BMI  < 25 (healthy)  25-30 (overweight)  30-35 (obese)  >35 (morbid obesity) | Ref.  7.93 (1.03, 14.82)  5.24 (-1.58, 12.06)  -2.5 (-9.27, 4.27) | Ref.  0.02  0.13  0.47 | Ref.  3.84 (0.27, 7.41)  1.56 (-1.98, 5.09)  -2.31 (-5.82, 1.21) | Ref.  0.04  0.39  0.20 |
| Alcohol Use | 0.88 (-2.08, 3.84) | 0.56 | 1.42 (-0.12, 2.96) | 0.07 |

‘Other’ race combines Asian, American Indian or Alaska Native, Native Hawaiian or other Pacific Islander and ‘none of the above’; CRP, C-reactive protein; SU, serum urate; CKD, chronic kidney disease, stage 3; RDCI, Rheumatic Disease Comorbidity Index; BMI, body mass index, kg/m^2^

|  | **Initial** | **24 weeks** | **48 weeks** | **72 weeks** |
| --- | --- | --- | --- | --- |
| **Overall HRQoL Measures** | | | | |
| **VR-6D**  Change from Initial  p-value  Effect Size (SMG)  N | 0.67 (0.12)  -  -  -  665 | 0.71 (0.12)  0.04 (0.10)  <0.001  0.32  665 | 0.70 (0.12)  0.04 (0.10)  <0.001  0.30  665 | 0.70 (0.12)  0.03 (0.10)  <0.001  0.29  665 |
| **EQ-5D-3L Index**  Change from Initial  p-value  Effect Size (SMG)  N | 0.71 (0.21)  -  -  -  665 | 0.76 (0.21)  0.05 (0.18)  <0.001  0.22  665 | 0.75 (0.22)  0.04 (0.19)  <0.001  0.19  665 | 0.74 (0.22)  0.03 (0.19)  <0.001  0.12  665 |
| **VR-12 Component Scores** | | | | |
| **VR-12 Physical (PCS)**  Change from Initial  p-value  Effect Size (SMG) | 37.3 (11.0)  -  -  - | 41.2 (11.3)  3.9 (9.8)  <0.001  0.35 | 40.9 (11.5)  3.6 (9.8)  <0.001  0.32 | 40.9 (11.5)  3.6 (10.4)  <0.001  0.32 |
| **VR-12 Mental (MCS)**  Change from Initial  p-value  Effect Size (SMG) | 51.6 (11.4)  -  -  - | 52.2 (10.8)  0.6 (9.7)  0.12  0.05 | 52.2 (10.8)  0.6 (10.4)  0.16  0.05 | 52.0 (10.9)  0.4 (10.8)  0.35  0.04 |
| **EQ-5D-3L Dimension Scores** | | | | |
| **Mobility**  Change from Initial  p-value  Effect Size (SMG) | 2.45 (0.50)  -  -  - | 2.59 (0.49)  0.14 (0.53)  <0.001  0.28 | 2.58 (0.50)  0.13 (0.53)  <0.001  0.25 | 2.54 (0.51)  0.09 (0.52)  <0.001  0.18 |
| **Self-Care**  Change from Initial  p-value  Effect Size (SMG) | 2.88 (0.33)  -  -  - | 2.88 (0.34)  0.00 (0.34)  0.91  0.00 | 2.87 (0.34)  -0.01 (0.33)  0.64  -0.02 | 2.86 (0.37)  -0.02 (0.36)  0.14  -0.06 |
| **Daily Activities**  Change from Initial  p-value  Effect Size (SMG) | 2.57 (0.54)  -  -  - | 2.65 (0.50)  0.08 (0.56)  <0.001  0.16 | 2.63 (0.53)  0.06 (0.60)  0.008  0.12 | 2.59 (0.53)  0.02 (0.59)  0.36  0.04 |
| **Pain**  Change from Initial  p-value  Effect Size (SMG) | 2.22 (0.57)  -  -  - | 2.40 (0.58)  0.17 (0.64)  <0.001  0.30 | 2.38 (0.58)  0.15 (0.63)  <0.001  0.27 | 2.36 (0.58)  0.14 (0.63)  <0.001  0.24 |
| **Mood**  Change from Initial  p-value  Effect Size (SMG) | 2.70 (0.51)  -  -  - | 2.68 (0.51)  -0.02 (0.48)  0.20  -0.05 | 2.69 (0.52)  -0.01 (0.49)  0.47  -0.03 | 2.67 (0.51)  -0.03 (0.48)  0.09  -0.06 |

**Supplemental Table 3:** Veterans Rand (VR)-12 (derived VR-6D) and EurolQol-5-Dimension-3-Level (EQ-5D-3L) scores in participants with data available at all time points receiving treat-to-target urate-lowering therapy at baseline, 24-, 48- and 72-wks; mean values shown (SD).
